# Effects of Thyroid Peroxidase Antibody on Maternal and Neonatal Outcomes in Pregnant Women in an Iodine-Sufficient Area in China

**DOI:** 10.1155/2016/6461380

**Published:** 2016-01-14

**Authors:** Xi Chen, Bai Jin, Jun Xia, Xincheng Tao, Xiaoping Huang, Lu Sun, Qingxin Yuan

**Affiliations:** ^1^Department of Endocrinology and Metabolism, The First Affiliated Hospital of Nanjing Medical University, Nanjing 210029, China; ^2^Department of Obstetrics, The First Affiliated Hospital of Nanjing Medical University, Nanjing 210029, China; ^3^Department of Endocrinology and Metabolism, The Affiliated Jiangsu Shengze Hospital of Nanjing Medical University, Suzhou 215000, China; ^4^Department of Nutrition, The First Affiliated Hospital of Nanjing Medical University, Nanjing 210029, China

## Abstract

*Purposes.* To evaluate the effects of thyroid peroxidase antibodies (TPOAb) on maternal and neonatal adverse outcomes in pregnant women.* Methods.* 208 pregnant women at 24–28 weeks were divided into two groups, TPOAb-positive and TPOAb-negative groups. Thyroid function and TPOAb were determined in all subjects until 12 months postpartum. Levothyroxine was supplemented to maintain euthyroid with periodical checking of thyroid functions. The prevalence of postpartum thyroiditis (PPT), placenta previa, placental abruption, premature rupture of membrane, postpartum haemorrhage, polyhydramnios, oligohydramnios, preterm birth, low birth weight, congenital hypothyroidism, and neonatal diseases were observed in two groups.* Results.* Of all women, 11.54% had a PPT. The prevalence of PPT was significantly higher in TPOAb-positive than TPOAb-negative group (42.31% versus 7.14%, *P* < 0.001), with 45.46% and 53.85% of PPT happening at 6 weeks postpartum in TPOAb-positive and TPOAb-negative groups. The incidence of polyhydramnios was significantly higher in TPOAb-positive than TPOAb-negative group (15.38% versus 2.74%, *P* = 0.02).* Conclusion.* Pregnant women with TPOAb-positive had increased risk of PPT, predominantly happening at 6 weeks postpartum. TPOAb was associated with increased incidence of polyhydramnios and the underlying mechanisms required further investigation. Earlier screening of thyroid function during pregnancy and postpartum was warranted in our region.

## 1. Introduction

Thyroid hormones are critical for mammalian life, including cardiovascular, kidney, and other organs, especially the neurological system [1–5]. Many changes occur in the thyroid gland, thyroid functions, iodine metabolism, and immune system during pregnancy [6, 7]. Pregnant women are particularly susceptible to hypothyroidism, which has been associated with a wide range of adverse outcomes, including miscarriage, placental abruption, preterm birth, fetal growth retardation, and impaired neuropsychological development of the offspring [8, 9].

Autoimmune thyroid disease is the most frequent cause of hypothyroidism in women of reproductive age. The prevalence of thyroid peroxidase antibody (TPOAb) in the general population of reproductive age is 10–20% [6]. The existence of TPOAb has been relevant to the possible development of thyroid dysfunction with the advancing gestation during pregnancy. Conflicting results on the impact of TPOAb on maternal and neonatal adverse outcomes have been published. A recent meta-analysis reported a significant association between thyroid autoantibodies and miscarriage [10]. In a study by Korevaar et al., TPOAb-positive was relevant to a 1.7-fold increased risk of premature [11]. However, another study did not observe the association between thyroid antibody positive and preterm birth [12]. Negro et al. reported a positive association between thyroid autoimmunity with preterm delivery and neonatal respiratory distress syndrome in euthyroid women [13], while no associations were found between preterm delivery and thyroid autoimmunity in the study by Bliddal et al. [14].

There still remains controversy regarding the screening strategies for thyroid functions and thyroid autoantibodies during prepregnancy, gestation, and postpartum [15–17]. Although guidelines recommended screening for thyroid diseases in the first trimester of pregnancy, there were still a large number of pregnant women with no screening for thyroid diseases in the first trimester in our country. A large proportion of women who were receiving prenatal care for the first time were already in the second trimester of pregnancy in most third-grade class-A hospital in our country such as our hospital, with thyroid function tests regarded as universal screening on the occasion. It is known that immune modulation that occurs during pregnancy would result in a decline of TPOAb levels with progression of pregnancy and sometimes antibodies became negative in the later gestation [18, 19]. This study was to observe the effects of TPOAb on adverse maternal and neonatal outcomes in pregnant women without earlier screening in an iodine-sufficient region in China, including the incidence of postpartum thyroiditis (PPT), placenta previa, placental abruption, premature rupture of fetal membrane (PROM), polyhydramnios, oligohydramnios, postpartum haemorrhage (PPH), preterm birth, fetal distress, low birth weight (LBW), and congenital hypothyroidism (CH).

## 2. Methods

### 2.1. Subjects

Initially, 401 singleton pregnant women who underwent regular examination at Jiangsu Province Hospital between January 2013 and July 2014 were enrolled in this study, with gestational age of 24–28 weeks, with checking for thyroid functions at least 3 times antepartum, and following up until 12 months postpartum. Pregnant women with a preexisting diagnosis of thyroid dysfunction, family history of thyroid diseases, assisted reproductive technology, and recognized system autoimmune diseases were excluded from this study. Among the initially screened, 185 were excluded for history of thyroid dysfunction, 3 for family history of thyroid diseases, 3 for assisted reproductive technology, and 2 for recognized system autoimmune diseases.

Data about maternal age, parity, and history of spontaneous abortion were collected by interrogation. Gestational age was calculated according to the first day of their last menstrual cycle (LMP) (for women with regular cycles), and/or ultrasonography for those with irregular cycles. Physical exams for all participants included weight, height, and systolic and diastolic pressure. All participants had been tested for gestational diabetes mellitus at 24–28 weeks through a 2-hour, 75 g oral glucose tolerance test. Physical examination of neonate was done to determine Apgar score, weight, and height.

Thyrotropin (TSH), free thyroxine (FT4), and urine iodine concentration (UIC) were measured at baseline and checked at 4-week interval during gestation. Levothyroxine (LT4) treatments were started in women with overt and subclinical hypothyroidism to maintain euthyroid antepartum and were withdrawn postpartum. Thus, subjects were divided into two groups, according to the results of TPOAb: TPOAb-positive and TPOAb-negative groups.

### 2.2. Measurements

3 mL of blood sample was obtained from each subject. Measurements of TSH, FT4, and TPOAb were carried out with the Cobas 6000 Centaur system (Roche Diagnostics, YZB/GEM 1815–2010, Mannheim, German), according to the instructions of the manufacturer (interassay CV <5.3%, intra-assay CV <6.4% for TSH and <7.8%, <7.1% for FT4). UICs were measured using the modified Sandell-Kolthoff reaction.

We used the ATA TSH criteria during pregnancy [16] (0.2–3 mIU/L, second trimester; 0.3–3, third trimester) and Roche's recommended range for FT4 [17] (9.81–17.26 pmol/L, second trimester; 9.12–15.71, third trimester) as the pregnancy-specific normal range.

Values of TPOAb >34 IU/mL were defined as being positive.

### 2.3. Definition of Abnormal Maternal Outcomes

Abnormal maternal outcomes were defined as follows. PPT is the occurrence of thyroid dysfunction in the first postpartum year in women who were euthyroid prior to pregnancy, including classical form, isolated thyrotoxicosis, and isolated hypothyroidism. In its classical form, transient thyrotoxicosis is followed by transient hypothyroidism with a return to the euthyroid state by the end of the initial postpartum year [16]. Placenta previa: the placenta is inserted partially or completely in the lower uterine segment, or the placenta partially or completely covers the internal orifice of the uterus. Placental abruption: the placental lining is partially or completely separated from the uterus of the mother before parturition. In PROM, the membrane of the amniotic sac and chorion is ruptured at least 1 h before the onset of labor [20]. Polyhydramnios and oligohydramnios: the amount of amniotic fluid exceeds 2000 mL and less than 400 mL, respectively. PPH: blood loss from the genital tract exceeds 500 mL within 24 hours after birth.

### 2.4. Definition of Abnormal Neonatal Outcomes

Abnormal neonatal outcomes were defined as follows: preterm birth: delivery occurring between 28 and 37 weeks of pregnancy; fetal distress: fetus with hypoxia during antepartum or intrapartum; LBW: fetal weight less than 2500 g; CH: neonates who had exhibited a positive screening for CH (TSH > 10 mU/L in heel blood) at birth subsequently confirmed as having a primary CH (TSH > 10 mU/L, FT4 < 12 pmol/L in venous blood). Neonatal diseases include neonatal haemorrhage, neonatal jaundice, neonatal thrombocytopenia, neonatal infections, and neonatal defects.

### 2.5. Statistical Analysis

Statistical analysis was performed using SPSS 19.0 software. Data were expressed as means ± standard deviation or numbers and percentages. Measurement data among different groups were carried by *t*-test. Serum TSH levels and UICs failed the normality test, presented as median with interquartile. Mann-Whitney *U* test was used for TSH and UICs in groups. TPOAb levels were presented as scatterplots with 25th, median, and 75th percentile values. The percentages of abnormal maternal and neonatal outcomes among different groups were carried by chi-squared test. *P* < 0.05 was considered to be statistically significant.

### 2.6. Ethics

The study was approved by the Ethics Committee of the First Affiliated Hospital of Nanjing Medical University. Written consent was obtained from the subjects individually. The purposes, the data collection procedures, and the benefits of the research were explained to them before obtaining their informed consent.

## 3. Results

### 3.1. Population Characteristics of Pregnant Women

**Figure 1:**
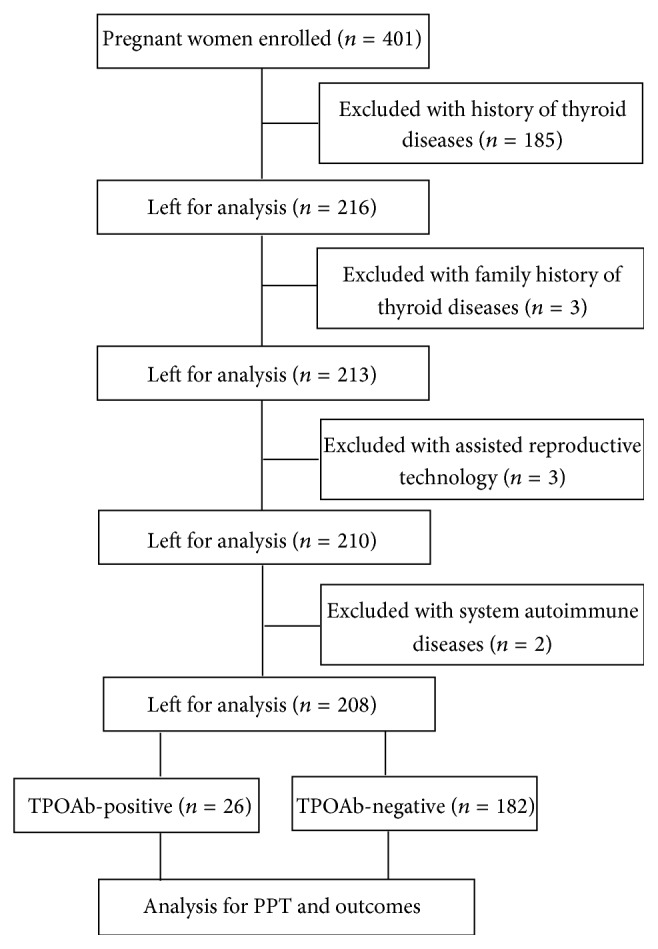
The flow diagram of the study process. PPT: postpartum thyroiditis.

The flow diagram of the study process was described in Figure 1. Finally, 26 women were in TPOAb-positive group and 182 women were in TPOAb-negative group.

The baseline characteristics of the study population were shown in Table 1. 208 pregnant women included 26 (12.50%) women in TPOAb-positive group and 182 (87.50%) women in TPOAb-negative group. There were no significant differences in age, BMI, gestational age, blood pressure, parity, history of spontaneous abortion, and blood glucose between two groups (*P* > 0.05).

Median urinary iodine concentration (MUIC) was 188 *μ*g/L in TPOAb-positive group and 202 *μ*g/L in TPOAb-negative group, indicating an iodine-sufficient population. There was no difference of UIC between two groups (*P* > 0.05).

### 3.2. Thyroid Functions of Women in TPOAb-Positive and TPOAb-Negative Groups Antepartum and Postpartum

In TPOAb-positive group, 2 (7.69%) women were with overt hypothyroidism and 4 (15.38%) women with subclinical hypothyroidism. Six (3.30%) women with overt hypothyroidism and 13 (7.14%) women with subclinical hypothyroidism were in TPOAb-negative group. Women with overt and subclinical hypothyroidism in both groups were treated with levothyroxine to maintain euthyroid. There has been no difference in the subjects treated with thyroxine in the two groups (*P* > 0.05).

TSH and FT4 were maintained in the normal range in both groups antepartum. There was no difference of thyroid functions between two groups antepartum (*P* > 0.05) (Table 2).

**Figure 2:**
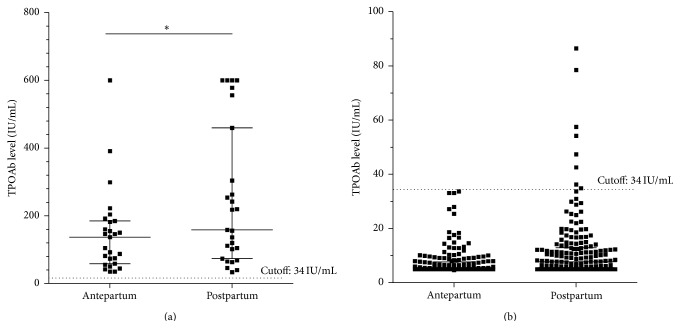
Changes of TPOAb levels antepartum and postpartum in TPOAb-positive and TPOAb-negative groups. (a) Changes of TPOAb levels antepartum and postpartum in TPOAb-positive group; (b) changes of TPOAb levels antepartum and postpartum in TPOAb-negative group. ^*∗*^ *P* < 0.01, TPOAb levels postpartum compared with antepartum in TPOAb-positive group.

As shown in Figure 2, the titres of TPOAb in TPOAb-positive group postpartum were much higher than the levels during pregnancy (*P* < 0.01). Eight women in the TPOAb-negative group became TPOAb-positive postpartum.

### 3.3. The Percentage of PPT in TPOAb-Positive and TPOAb-Negative Groups

Of all women, 11.54% (24/208) had a PPT. The prevalence of PPT was significantly higher in TPOAb-positive than in TPOAb-negative group (42.31% versus 7.14%, *P* < 0.001), with 45.46% (5/11) and 53.85% (7/13) of PPT happening at 6 weeks postpartum in TPOAb-positive and TPOAb-negative groups, respectively. 63.64% (7/11) of cases presented as classical form in TPOAb-positive group and 61.54% (8/13) of cases presented as isolated thyrotoxicosis in TPOAb-negative group (Table 3).

### 3.4. Adverse Maternal Outcomes in TPOAb-Positive and TPOAb-Negative Groups

As depicted in Table 4, the incidence of polyhydramnios was significantly higher in TPOAb-positive than in TPOAb-negative group (15.38% versus 2.74%, *P* = 0.02). The gestational age at delivery was comparable between the two groups (*P* > 0.05). The rate of placenta previa, placental abruption, PROM, oligohydramnios, and PPH had no difference between the two groups (*P* > 0.05).

### 3.5. Neonatal Outcomes in TPOAb-Positive and TPOAb-Negative Groups

Neonatal outcomes in two groups were compared in Table 5, and no significant difference in the incidence of preterm birth, fetal distress, LBW, neonatal diseases, and CH was observed between the two groups (*P* > 0.05). Apgar score at 1 and 5 min, neonate weight, and height were comparable in the two groups (*P* > 0.05).

## 4. Discussion

In the current study, we investigated the effects of TOPAb on maternal and neonatal outcomes in pregnant women in the second trimester. It was found that pregnant women who are TPOAb-positive had increased risk of PPT, which predominantly happened at 6 weeks postpartum. The presence of TPOAb was associated with increased incidence of polyhydramnios.

Prior research demonstrated a large variation in the prevalence of PPT, ranging from 1.1 to 16.7% [21], with higher prevalence in high risk groups such as women with type 1 diabetes mellitus, a family history of thyroid diseases, or other autoimmune diseases. In the present study, PPT occurred in 11.54% of women, including 42.31% of women in TPOAb-postive group and 7.14% in TPOAb-nagtive group. Possible reasons for the different incidence between the studies could be related to the different assay methods, iodine intake, postpartum testing times, and environment factors. Consistent with previous studies, TPOAb-positive women had much higher prevalence of PPT compared to TPOAb-negative women. Although strong correlation between TPOAb and PPT was observed, the underlying mechanism was not fully clear. The rebound action of the immune system in the postpartum period could be one of the reasons [22, 23]. The immune modulation that occurs during pregnancy would result in a decline of TPOAb levels with progression of pregnancy. The levels of TPOAb postpartum can restore to prepregnancy levels, leading to the increased destruction of thyroid gland. Furthermore, it had reported that TPOAb is related to thyroid injury and the IgG1 subtype of TPOAb may play a vital role in the damage of the thyroid in PPT [24].

The onset time of PPT has not been explicitly reported in the literature. Almost half of PPT happened at 6 weeks postpartum in both groups in our study, although it is known that the peak TPOAb in the postpartum can occur up to 6 months postpartum [19]. It is suggested that thyroid function tests should be performed at approximately 6 weeks postpartum especially in TPOAb-positive women. The presenting forms of PPT included classical form, isolated thyrotoxicosis, and isolated hypothyroidism. In this study, 63.64% of PPT presented as classical form in TPOAb-positive group and 61.54% presented as isolated thyrotoxicosis in TPOAb-negative group. The titres of TPOAb in TPOAb-positive group postpartum were much higher than the levels during pregnancy in our study. With the higher levels of TPOAb, much more injury of the thyroid gland would happen, with much more thyroid hormones released (the transient thyrotoxicosis) followed by hypothyroidism. Eight women in the TPOAb-negative group became TPOAb-positive postpartum; 5 in 8 presented with PPT. Compared with TPOAb-positive group, the levels of TPOAb in these women were lower and the damage to thyroid gland might be slighter. The interim time from thyrotoxicosis to hypothyroidism might be longer, presenting as isolated thyrotoxicosis in the first postpartum year. The underlying clear mechanisms need further study. Considering that PPT may become permanent in the postpartum or portend thyroid failure in later years [25], close follow-up is required especially in patients who are TPOAb-positive in order to identify the cases developing permanent thyroid dysfunction.

While some researches demonstrated that TPOAb results in maternal and neonatal complications such as miscarriage, preterm birth, and LBW, this had not been reported by others. In the present study, TPOAb was not found to be associated with placenta previa, placental abruption, PROM, oligohydramnios, PPH, premature delivery, fetal distress, LBW, neonatal diseases, and CH. Chen et al. reported that TPOAb was associated with PROM and LBW; no association was found with other outcomes, such as gestation diabetes, gestation hypertension, placenta previa, placental abruption, fetal distress, and preterm birth [20]. The reasons for the negative results of the above outcomes in our study would be the later time of thyroid function tests. It is possible that some subjects of the TPOAb-negative group may indeed have been TPOAb-positive in the earlier pregnancy owing to the decline of TPOAb levels with progression of pregnancy. Interestingly, we found that women who are TPOAb-positive had significantly higher rate of polyhydramnios compared to TPOAb-negative group. Common causes of polyhydramnios are gestational diabetes, fetal anomalies with disturbed fetal swallowing of amniotic fluid, fetal infections, and other, rarer causes [26]. The observed significance had no relationship with the blood glucose due to the comparative levels of blood glucose in two groups. Underlying factors that regulate the association between TPOAb and polyhydramnios warrant further investigation. Moreover, our results need to be validated with larger number of subjects from various medical centers. While American Thyroid Association (ATA) commented on the lack of evidence needed to make any recommendations for the screening of thyroid autoantibodies in pregnancy [16]. Given the importance of the potential negative effects of TPOAb on polyhydramnios, which is associated with increased adverse obstetric and neonatal outcomes [26, 27], we recommended that screening for TPOAb in pregnancy should be performed so far.

The adverse maternal and fetal effects linked with undiagnosed and untreated overt thyroid dysfunction in pregnant women have been clearly depicted in previous literature and guidelines [16, 28]. It is well accepted that overt and subclinical hypothyroidism have a deleterious impact on pregnancy. Considering the ethics, women with overt and subclinical hypothyroidism in the both groups were treated with levothyroxine to maintain euthyroid in our study. There has been no difference in the subjects treated with thyroxine in the two groups. Furthermore, maternal iodine deficiency may result in adverse maternal and neonatal outcomes, including miscarriage, preterm birth, and impaired neurodevelopment of offspring [7]. Therefore, we performed our study in an iodine-sufficient population to avoid the influence of iodine nutrition on the observed outcomes.

Various reports regarding the influence of TPOAb on the impaired neuropsychological development of offspring have been published. Li et al. reported that lower motor and intellectual development of children at 25–30 months of age was associated with elevated TPOAb of women at 16–20-week gestation [29]. A study from Ghassabian et al. implied that the elevated titers of TPOAb during pregnancy impact children's risk of problem behavior, in particular attention deficit/hyperactivity [30]. Infant outcomes are of greater clinical significant concerns, such as cognitive and psychomotor development of offspring. Therefore, with the extension of follow-up time, we would obtain much more data to clarify the impact of TPOAb on the neuropsychological performance, including motor and intellectual development.

In conclusion, pregnant women who are TPOAb-positive had increased risk of PPT and polyhydramnios. We recommended that screening of thyroid function should be performed at earlier pregnancy and at approximately 6 weeks postpartum especially in TPOAb-positive women. The potential effects of TPOAb on polyhydramnios and neurodevelopment of offspring warrant further investigation.

## Figures and Tables

**Table 1:** Population characteristics of TPOAb-positive and TPOAb-negative women during pregnancy.

	TPOAb-positive (*n* = 26)	TPOAb-negative (*n* = 182)	*P*
Maternal age (years)	29.15 ± 4.10	29.46 ± 4.08	0.71
BMI (kg/m^2^)	21.45 ± 3.45	21.13 ± 2.36	0.55
Gestational age (weeks)	25.24 ± 1.26	24.99 ± 1.41	0.73
Parity			0.57
Nulliparity	21 (80.77%)	154 (84.62%)	
Multiparity	5 (19.23%)	28 (15.38%)	
History of spontaneous abortion	3 (11.54%)	8 (4.40%)	0.14
Blood pressure			
Systolic pressure (mmHg)	118.24 ± 23.15	116.51 ± 12.26	0.62
Diastolic pressure (mmHg)	74.43 ± 6.71	76.31 ± 7.53	0.37
FBG (mmol/L)	4.69 ± 0.48	4.78 ± 0.57	0.56
PBG1h (mmol/L)	8.17 ± 1.65	8.22 ± 1.93	0.86
PBG2h (mmol/L)	7.04 ± 1.46	7.30 ± 1.74	0.53
MUIC (*μ*g/L)	188	202	0.23

TPOAb, thyroid peroxidase antibody; BMI, Body Mass Index; MUIC, median urine iodine concentration.

FBG, fasting blood glucose; PBG1h, 1 h plasma blood glucose; PBG2h, 2 h plasma blood glucose.

Data were presented as mean ± standard deviation (SD) or *n* (%).

*P*, compared TPOAb-positive with TPOAb-negative groups.

**Table 2:** Thyroid functions of women in TPOAb-positive and TPOAb-negative groups antepartum.

	TPOAb-positive (*n* = 26)	TPOAb-negative (*n* = 182)	*P*
TSH (mIU/L)			
24–28 W	4.05 (0.77–11.36)	3.01 (0.47–12.31)	0.07
28–32 W	2.38 (0.32–10.45)	2.34 (0.33–9.67)	0.56
32–36 W	2.24 (0.36–6.38)	2.83 (0.31–8.62)	0.22
36–40 W	2.13 (0.21–5.04)	2.92 (0.22–5.20)	0.34
FT4 (pmol/L)			
24–28 W	11.24 ± 2.37	11.15 ± 2.22	0.17
28–32 W	12.02 ± 2.30	11.39 ± 1.68	0.23
32–36 W	11.68 ± 1.73	11.62 ± 1.71	0.26
36–40 W	12.26 ± 4.62	11.71 ± 1.81	0.18

TPOAb, thyroid peroxidase antibody.

TSH were presented as median with interquartile; FT4 were presented as mean ± standard deviation (SD).

*P*, compared TPOAb-positive with TPOAb-negative groups.

**Table 3:** Onset time and presenting form of PPT in TPOAb-positive and TPOAb-negative groups.

	TPOAb-positive (*n* = 26)	TPOAb-negative (*n* = 182)
PPT, *n* (%)	11 (42.31%)^*∗*^	13 (7.14%)
Postpartum time of onset		
6 weeks postpartum, *n* (%)	5 (45.46%)	7 (53.85%)
3 months postpartum, *n* (%)	2 (18.18%)	3 (23.08%)
6 months postpartum, *n* (%)	3 (27.27%)	2 (15.38%)
12 months postpartum, *n* (%)	1 (9.09%)	1 (7.69%)
Presenting form		
Classical form, *n* (%)	7 (63.64%)	2 (15.38%)
Isolated thyrotoxicosis, *n* (%)	1 (9.09%)	8 (61.54%)
Isolated hypothyroidism, *n* (%)	3 (27.27%)	3 (23.08%)

PPT, postpartum thyroiditis.

^*∗*^ *P* < 0.001, the percentage of PPT between TPOAb-positive and TPOAb-negative groups.

**Table 4:** Maternal outcomes in TPOAb-positive and TPOAb-negative groups.

Pregnancy outcomes	TPOAb-positive (*n* = 26)	TPOAb-negative (*n* = 182)	*P*
Gestational age at delivery (weeks)	38.24 ± 1.26	38.99 ± 1.41	0.73
Mode of delivery			0.54
Vaginal	15 (57.69%)	94 (51.65%)	
Cesarean	11 (42.31%)	88 (48.35%)	
Placenta previa	0 (0%)	1 (0.55%)	1
Placental abruption	0 (0%)	1 (0.55%)	1
PROM	3 (11.54%)	11 (6.04%)	0.39
Polyhydramnios	4 (15.38%)	5 (2.74%)	0.02
Oligohydramnios	2 (7.69%)	22 (12.09%)	0.74
PPH	2 (7.69%)	10 (5.49%)	0.21

PROM, premature rupture of fetal membrane; PPH, postpartum haemorrhage.

Data were presented as *n* (%) or mean ± standard deviation (SD).

The percentages of abnormal maternal outcomes between the two groups were compared by Fisher's exact test; gestational age at delivery between the two groups was carried by *t*-test.

**Table 5:** Neonatal outcomes in TPOAb-positive and TPOAb-negative groups.

Neonatal outcomes	TPOAb-positive (*n* = 26)	TPOAb-negative (*n* = 182)	*P*
Sex of neonate (boy, %)	12 (46.15%)	96 (52.75%)	0.54
Neonate weight (g)	3369.05 ± 249.31	3404.82 ± 461.15	0.73
Neonate height (cm)	49.89 ± 0.24	50.02 ± 0.90	0.91
Apgar score at 1 minute	9.95 ± 0.22	9.88 ± 0.74	0.67
Apgar score at 5 minutes	10	9.96 ± 0.28	0.55
Premature delivery	2 (7.69%)	9 (4.95%)	0.63
Fetal distress	2 (7.69%)	9 (4.95%)	0.63
LBW	0	5 (2.75%)	1
Neonatal diseases	2 (7.69%)	9 (4.95%)	0.63
Congenital hypothyroidism	1 (3.85%)	1 (0.55%)	1

LBW, low birth weight.

Data were presented as *n* (%) or mean ± standard deviation (SD).

The percentages of abnormal neonatal outcomes between the two groups were compared by Fisher's exact test; neonate weight, height, and Apgar scores between the two groups were carried by *t*-test.
